# The Alleged Increase of Insanity

**Published:** 1893-09

**Authors:** C. S. W. Cobbold

**Affiliations:** Formerly Medical Superintendent of the Earlswood Asylum


THE BRISTOL

Medico-Chirurgical Journal.

SEPTEMBER, 1893.

### THE ALLEGED INCREASE OF INSANITY.

BY

C. S. W. Cobbold, M.D. Würz., F.R.C.P. Ed.

*Formerly Medical Superintendent of the Earlswood Asylum.*

The earlier part of this paper will necessarily deal with figures and statistics; later on I propose to speak of some of the causes which are tending to increase or diminish insanity.

Table I.

ENGLAND AND WALES.

*Registered as Insane.*

| | | | | | |
|---|---|---|---|---|---|
| January 1st, | 1859 | ... | ... | ... | 36,762 |
| ,, | 1869 | ... | ... | ... | 53,177 |
| ,, | 1879 | ... | ... | ... | 69,885 |
| ,, | 1889 | ... | ... | ... | 84,340 |
| ,, | 1890 | ... | ... | ... | 86,067 |
| ,, | 1891 | ... | ... | ... | 86,795 |
| ,, | 1892 | ... | ... | ... | 87,848 |
| ,, | 1893 | ... | ... | ... | 89,822 |

According to Table I., the official returns show that the number of persons registered as insane in England and Wales was considerably more than doubled during the thirty years between 1859 and 1889. In 1859 the total number was 36,762; in 1889 it had risen to 84,340. Since then the number has continued to rise; and on the 1st of January, 1893, it had reached 89,822.

The population has not increased at anything like the same rate; so that at first sight it would seem that the question, "Is insanity increasing?" is definitely answered in the affirmative. But the matter is not so simple: we must first attach a definite meaning to the expression, "Increase of insanity." If by that expression we mean increase in the proportion of officially-recognised lunatics to the population, then we are right in saying that insanity is increasing, or, rather, that it has very largely increased during recent years. If, on the other hand, we inquire whether *fresh cases of insanity* arise with greater frequency now (in proportion to population) than they did a few years ago, the question is far more difficult to answer. And this, surely, is the point which it is most important to consider with a view to the future welfare of the community. If we find that fresh cases do not occur with markedly increased frequency, we may feel with some degree of confidence that there is no danger of the race becoming permeated with insanity to such an extent as to threaten social order.

TABLE II.

ENGLAND AND WALES.

*Ratio (per 10,000) of "Admissions" to Population.*

| | All Admissions. | Omitting Admissions to County and Borough Asylums. |
|---|---|---|
| 1869 ... ... | 4·71 | |
| 1878 ... ... | 5·33 | |
| 1879 ... ... | 5·16 | |
| 1882 ... ... | 5·17 | ... ... 1.07 |
| 1883 ... ... | [1] 5·43 | ... ... 1.00 |
| 1884 ... ... | 5·31 | ... ... 0.88 |
| 1885 ... ... | 4·90 | ... ... 0.82 |
| 1886 ... ... | 4·93 | ... ... 0.79 |
| 1887 ... ... | 5·14 | ... ... 0.89 |
| 1888 ... ... | 5·25 | ... ... 0.86 |
| 1889 ... ... | 5·29 | ... ... 0.90 |
| 1890 ... ... | 5·63 | ... ... 0.86 |
| 1891 ... ... | 5·74 | ... ... 0.90 |
| 1892 ... ... | 5·83 | ... ... 0.92 |

[1] Or 5.15, allowing for transfer in Lancashire of 668 above average.

With reference to the proportion of new cases of insanity to population, I would ask attention to Table II., which deals with new admissions into asylums, &c. These, in the great majority of instances, are fresh cases of insanity. We see here that the increase is very gradual and slight. For the twelve years bracketed from 1878 to 1889 inclusive, there was absolutely no increase whatever in the proportion of new admissions to population. The last three years upon the table do show a somewhat increased rate, and it will be interesting to observe whether this higher rate will be maintained, or whether (being due to temporary causes only) it will sink again, as it has done before, notably in 1885 and 1886. With regard to the years 1890 and 1891, the Commissioners remark in their last Report but one that "the admissions of the last two years have been swelled by the reception from workhouses of a considerable number of patients previously classed therein as of unsound mind," but which still count as fresh admissions to asylums. I prefer to accept the Commissioners' explanation rather than attribute the slight increase since 1890 to the influenza or to any change of system introduced by the County Councils.

The statistics I have referred to up to the present include *all* registered cases, both private and pauper; but as the pauper lunatics in this country are immensely the more numerous, they practically swamp the lunacy statistics. It is easy to understand that in lunacy, as well as in other statistics, the so-called "classes" are swamped by the "masses." The opening of a new pauper lunatic asylum always has a most marked influence upon the statistics of the year. It nearly always happens that a number of imbecile or partly-demented paupers, who have hitherto been kept quietly in the workhouses, are at once certified and drafted off to the new county or borough asylum, where they count as new "admissions." This makes it appear in the statistics that a sudden accession of fresh cases of insanity has taken place, whereas nothing of the kind has occurred. A very good instance of this is seen in Table II. for the year 1883. During that year a large amount of fresh accommodation for pauper lunatics became available in the County Palatine of Lancaster. As a result of this, paupers to the number of 668 above the annual average for that county were certified and placed in the Lancashire asylums; but no one believes that new cases of insanity were much more common than usual that year among Lancashire paupers. The influence of this rearrangement is seen in the table. The ratio for 1883 would stand at 5.15, instead of 5.43, if it were not for the excess of 668 transfers above the average in Lancashire in that particular year.

It is quite a question whether the assiduous gathering of insane paupers, and of chronic and paralytic cases above the pauper class, into our comfortable county and borough asylums is not the *sole* cause of any apparent increase in the ratio of new cases to population. There are no means of proving absolutely whether this is so or not, but the instance I have given (of the year 1883) points strongly in that direction. There is no doubt that whenever comfortable asylum accommodation is provided at the public expense, people take advantage of it (as Dr. Clouston recently wrote) to "get rid of troublesome dotards and paralytics."

"Private" patients (*i.e.* those who pay by themselves or their friends for their maintenance) are not subject to the same wholesale movements and rearrangements as paupers. The statistics with regard to private patients only, would therefore be a safer guide as to the increase or otherwise in the proportion of new cases to population. Unfortunately, however, these figures are not given by the Lunacy Commissioners. In order to supply this deficiency, I have myself made some calculations, and the results are given in the third column of Table II. My figures refer to new admissions into all kinds of asylums, omitting only admissions to county and borough asylums. The result shows that the proportion of admissions to private asylums, registered hospitals, "single care," &c., has been actually lower during the last nine years than it was in 1882 and 1883, although during that period the proportion of families in comfortable circumstances is known to have largely increased.

The decrease is certainly not due to any scarcity of accommodation, because a large new registered hospital has been developed at Virginia Water, and other hospitals receiving only private patients have increased their accommodation during the years in question. I do not contend that this table absolutely proves a diminishing proportion of new private cases, because there are considerable sources of error which must not be overlooked. The chief of these lies in the fact that some pauper patients are admitted into private asylums, and that private patients (at low rates of payment) are admitted to the county and borough asylums in larger numbers than formerly. Still I *do* believe that these figures afford a very fair criterion as to what is actually taking place, inasmuch as I think the sources of error about counterbalance one another. For example, the increase in the proportion of families of independent means tends to increase the proportion of admissions in this column; the attraction of private patients to pauper asylums tends to lower it, and the two circumstances probably about counteract one another. There appears then to be satisfactory evidence that the proportion of new cases to population has not very materially increased during the past ten or twelve years, and yet we are face to face with the figures in Table I., which show an enormous increase during the past thirty or forty years in the total number of registered lunatics. I shall endeavour briefly to explain this apparent discrepancy.

It may appear paradoxical to say so, but the real cause of the increase in the numbers of our lunatics is the great reform which the last half-century has witnessed in the care and treatment of the insane. The humane treatment of recent years has led to a great increase in the average length of life of the insane: the old cases live on now instead of dying, as they would have done under the old system of neglect. New cases are added annually at about the same rate as before, and the result is an "accumulation" of lunatics. It is to this cause (accumulation), taken together with the building and filling of commodious county and borough asylums, and the more careful certification and registration of lunatics (all of which are part of the great reform), that we must ascribe the enormous rise of numbers since the year 1859. During the last few years the same causes have continued in operation, but in a much less degree. Matters have almost found their level. As a result, we see that the increase during the last ten or fifteen years has been far more gradual, and is very little more than proportionate to the growth of the population.

TABLE III.

ENGLAND AND WALES.

*Ratio (per 10,000) of Registered Insane to Population.*

| | All Insane. | "Private" cases only. |
|---|---|---|
| January 1st, 1859 | 18.67 | 2.38 |
| " 1869 | 23.93 | 2.61 |
| " 1879 | 27.54 | 2.97 |
| " 1883 | 28.83 | 2.89 |
| " 1884 | 29.17 | 2.91 |
| " 1885 | 29.28 | 2.85 |
| " 1886 | 29.12 | 2.83 |
| " 1887 | 29.07 | 2,81 |
| " 1888 | 29.37 | 2.77 |
| " 1889 | 29.65 | 2.80 |
| " 1890 | 29.92 | 2.81 |
| " 1891 | 29.85 | 2.82 |
| " 1892 | 29.88 | 2.82 |
| " 1893 | 30.21 | 2.76 |

Looking at Table III., we see that the ratio of the insane to the population has fluctuated during the last ten years, without showing any great increase. In the years 1886, 1887, and 1891 there was even a slight decrease. It is believed that the slight increase during the ten years is not greater than can be accounted for by the gathering in and registering of insane paupers, which has before been alluded to. It is well known that, as part of the great reform in our lunacy system, a greater proportion of imbeciles and chronic lunatics are now certified and registered as insane than was formerly the case. The control of our county asylums by County Councils, the growth of education and consequent raising of the normal standard of intellect, the Government grant in aid of paupers in asylums, have all of them their influence in this direction.

The *whole of the increase* in the proportion of the insane to the population during the past fourteen years has taken place among the *pauper* class. That this is so is evident on referring to the third column in Table III. This gives the proportion for "private" cases only. During the fourteen years the figures have fluctuated somewhat irregularly, but on the whole they have slightly declined from 2.97 in 1879 to 2.76 in 1893. This absence of increase is all the more significant and satisfactory when we reflect that the proportion of families who are independent, and in a position to pay for the maintenance of their insane relatives as "private" patients, is proved by income-tax statistics to be steadily upon the increase. Dr. Clouston very pertinently puts the question: "If insanity, as a disease, is really increasing, why should the increase be confined to those who get treated at the public expense?"

I believe I have shown that though lunatics are "accumulating," new cases of lunacy are not materially more frequent in proportion to population than they have been in past years. This is the view which is taken by the Commissioners in Lunacy, who are certainly in the best position for arriving at the truth in this matter. This view has also been recently strongly supported by Dr. Clouston, of the Royal Edinburgh Asylum, in his last annual report, from which I have already quoted. His figures refer more especially to Scotland and Ireland; and it is interesting to observe that as the great wave of lunacy reform was later in those countries than in England and Wales, so the lunacy statistics follow the same lines, but are some years behind. The reform being later, the bulk of the apparent increase in lunacy was also later. Since I wrote this last passage the matter, so far as regards Ireland, has been referred to by Mr. A. J. Balfour in the House of Commons in the following terms: "Lunatics have increased because lunatics are now looked after. Our knowledge of them, our registration of them, shows a larger number, because in the century to which the hon. gentleman looks as the Golden Age of Ireland the lunatics were tied to posts or allowed to stray untended in the fields."[1]

In the January number of *The Fortnightly Review* Mr. W. J. Corbet makes an attack upon the view taken by the Commissioners in Lunacy, and which is advocated in the present paper. I fail to see that Mr. Corbet in any way shakes the position

[1] *Standard*, April 22nd, 1893.

taken by the Commissioners. He says that their arguments "have been a long time in use," and "have grown stale and of little or no account." That the same view has been held by the Commissioners for some years past affords no presumption whatever that it is a wrong one; that the arguments are "of little or no account" is Mr. Corbet's statement, which, in my opinion, he fails to substantiate.

The Commissioners in their Report for 1892 still point to the "accumulation of chronic cases in the wards of county and borough asylums" as the chief cause of the annual increase in the total number of lunatics under care. They further say: "The confidence which the public has learnt to place in the management of these institutions (county and borough asylums) has no doubt conduced to the placing in them of many persons, also incurable, but above the status of paupers, their relatives reimbursing the Guardians the cost of their maintenance."

I propose now to touch upon some of the causes which are operating at the present time in the direction of increasing or diminishing insanity in our midst.

Any inquiry as to what causes are tending to-day to increase the numbers of new cases of insanity must necessarily be of a very speculative nature, and afford ample scope for wide differences of opinion. Such an inquiry should include the question as to what influences are acting to-day in a contrary direction, and are tending to diminish lunacy in the population. I will here mention a few considerations which bear upon such an inquiry, but cannot attempt to discuss the question exhaustively.

Civilisation (with its attendant bustle, hurry, competition, and brain-struggle) is constantly accused as being the chief factor in whatever increase in insanity may be taking place. We are told that a certain percentage of lunatics is one of the prices we have to pay for the advantages of civilisation. It has even been said that where there is no civilisation, there is no insanity. With regard to uncivilised communities, we have certainly no lunacy statistics to guide us. (This will be regarded by some as an unmixed blessing.) This affords no proof, however, that cases of insanity do not occur among savage races,

It is true that barbarous nations do not support costly asylums, in which lunatics and failures of development in the form of idiots are carefully nurtured and kept alive; but there is no proof that such beings are not produced. The savage has far readier methods of dealing with an insane brother: he may exalt him into a deity and worship him; he may believe him to be a devil to be propitiated; or, taking the view that such a being ought not to live, he may adopt some very summary method of giving effect to his opinion, and may even come to regard a case of dementia as a cogent argument in favour of cannibalism.

I make bold to dispute the statement that there is no madness among savages, and I claim to have shown earlier in this paper that new cases are not materially increasing in a typical civilised community (England and Wales).

No doubt we live too rapidly for perfect mental health, and "it is the pace that kills": the worry of business is more wearing than the quiet labour of a rustic community; but, on the other hand, the unused brain degenerates as surely as the overwrought one, though with a different train of symptoms. The rush and roar of a fevered civilisation lead to the overthrow of many weak mental constitutions, but they also favour the development of mental powers which may be sought in vain among simple village communities. Still overpressure in education (with which may be included the competitive examination system) must certainly be regarded as an increasing cause of mental breakdown.

Alcoholic intemperance is of course a fertile cause of madness: it is immensely prevalent in newly-civilised communities; but I believe we are justified in believing that alcoholism is slowly on the decrease in this country, owing chiefly to the spread of education and consequent self-control among the masses.

Heredity stands always in the official statistics at the head of the list of "causes of insanity." Whether its influence is increasing or decreasing is difficult to decide; but there is no doubt that it is far more potent than any other cause. Among barbarous communities it is probable that a person who had once become lunatic would have few opportunities of propagating either his species or his lunacy. Under our reformed lunacy system, lunatics are carefully tended, treated, cured, and let loose upon the world with every opportunity of transmitting the taint of insanity to posterity. We seem here to be face to face with an appalling danger to the community. During last year only (1892) no less than 11,201 persons were discharged from lunatic asylums: 6,670 of these were said to have "recovered"; many of them were already married; of the unmarried, many will no doubt marry and become parents. Are our splendidly-managed hospitals for the insane thus to become centres from which the taint of hereditary mental disease is to descend to posterity? No doubt this must be so to a great extent; but I venture to think that we may derive some comfort from the tendency of healthy stock to prevail over diseased stock. The healthy strain being the stronger, tends, under favourable conditions, to perpetuate itself, and the diseased strain, under equally favourable conditions, tends to lose its influence for harm.

Another cause of insanity which has only become operative during the last few years is influenza. There is much difference of opinion among asylum physicians as to the amount of insanity which may fairly be ascribed to influenza; but all will probably agree that it has directly caused a not inconsiderable number of cases, while none, I think, will maintain that its influence has been sufficiently great to cause any marked increase in statistics such as are dealt with in this paper.

It is only right to recognise that there are also influences at work which tend to the diminution of new cases of insanity. Among these education and sanitation are regarded as the most important. The properly educated mind tends to be a well-balanced mind; its powers of self-control are better developed, and its judgment has been cultivated. These qualities tend to keep it in a healthy channel, to guard its owner against excesses of all kinds, including alcoholic intemperance and overwork.

Sanitation is of course specially calculated to promote the physical health of the community, but in doing this it benefits the mental health in an almost equal degree. Good bodily health is of course the best foundation for a healthy condition of mind.

One great result of sanitation lies in the fact that it has enormously decreased the amount of fever, and consequently of post-febrile insanity, among the masses. In the old days, when typhus and typhoid were rife (and undistinguished from one another), post-febrile insanity was as common as it now is comparatively rare.

In conclusion, I submit that civilisation and education (or rather their accompaniments) must at present be reckoned as potent causes of insanity and other nerve diseases. But civilisation and education are good in themselves; the evil which they cause is no necessary sequel, but is due to faulty systems and to errors which have grown up with them, though they form no part of them, and may in time be separated from them. The good which they do is constantly upon the increase, and more than counterbalances the harm which is unfortunately incidental to their development.

